# Midwives' Evaluation of a Neonatal Resuscitation in High- and Low-Resource Settings

**DOI:** 10.3389/fped.2021.644308

**Published:** 2021-03-09

**Authors:** Francesco Cavallin, Serena Calgaro, Martina Borellini, Margherita Magnani, Greta Beltramini, Amir Hussein Abubacar Seni, Bonifacio Rodriguez Cebola, Ana Nicolau Tambo, Giovanni Putoto, Daniele Trevisanuto

**Affiliations:** ^1^Independent Statistician, Solagna, Italy; ^2^Doctors With Africa Collegio Universitario Aspiranti Medici Missionari, Beira, Mozambique; ^3^Department of Woman's and Child's Health, University of Padua, Padua, Italy; ^4^Department of Pediatrics, Beira Central Hospital, Beira, Mozambique; ^5^Doctors With Africa Collegio Universitario Aspiranti Medici Missionari, Padua, Italy

**Keywords:** midwife, neonatal resuscitation, video-recording, Mozambique, Italy

## Abstract

**Aim:** To assess midwives' evaluation of a real-life neonatal resuscitation and their opinion on importance of resuscitation interventions.

**Methods:** Multicenter, multi-country study.

**Setting:** Beira Central Hospital (Mozambique) and Azienda Ospedale-Università di Padova (Italy).

**Subjects:** Sixteen Mozambican midwives and 18 Italian midwives.

**Interventions:** Midwives' assessment was evaluated by using a predefined score, which graded each resuscitation intervention (0–2 points) and summed to a total score for each step (initial steps, bag-mask ventilation, and chest compressions). All scores were compared with referral scores given by two expert neonatologists.

**Results:** Both Mozambican and Italian midwives overestimated their performance regarding of initial steps taken during resuscitation, chest compressions, high-oxygen concentrations (*p* < 0.01), and underestimated the importance of stimulation (*p* < 0.05). Mozambicans overestimated suctioning (*p* < 0.001). Participants agreed with experts about the importance of equipment preparation, using a warmer, drying the newborn, removing wet linen and heart rate assessment.

**Conclusion:** Mozambican and Italian midwives overestimated the performance of a real-life neonatal resuscitation, with heterogeneous evaluation of the importance of several aspects of neonatal resuscitation. These findings may be useful for identifying educational goals.

## Introduction

Globally, 2.5 million newborns died in 2018, accounting for 47% of under-5 child mortality. Around 75% of neonatal deaths occurred during the first week of life, including 1 million newborns who died within the first 24 h (1, 2). The reduction of neonatal mortality in low-income countries represents a global health priority, with Sub-Saharan Africa displaying the highest neonatal mortality rate worldwide (28 deaths per 1,000 live births) (1, 2).

About one out of four neonatal deaths is related to intrapartum-related events (2), hence interventions focusing on perinatal period can contribute to the reduction of neonatal mortality. Education in neonatal resuscitation for all health workers who are involved in the management of the newborn at birth plays an important role in improving neonatal survival (3). However, implementation of training programs in neonatal resuscitation resulted in limited impact on technical and non-technical skills, and clinical outcomes in low-income settings (4–6). While great efforts have been dedicated in developing neonatal resuscitation training programs (7, 8), understanding the best approach to ensure and maintain adequate skill levels requires further research (9). In high-income settings, high-frequency/low-dose training combined with performance-debriefings using video recordings showed some benefits in clinical outcomes as well as adherence to algorithm of neonatal resuscitation (10).

Beyond advantages in performance-debriefings, video recordings may be useful in understanding how healthcare providers evaluate performance of their peers during actual resuscitation procedures. We speculate that knowledge on this aspect may help trainers in identifying areas of improvement and educational goals. This study aimed to assess how Mozambican and Italian midwives evaluated performance of their peer during real-life neonatal resuscitations. In addition, their opinions on importance of resuscitation interventions were investigated.

## Materials and Methods

### Study Design

This multicenter, multicountry study investigated (i) how Mozambican and Italian midwives assess a real-life video on neonatal resuscitations and (ii) their opinion on importance of resuscitation interventions.

### Participants and Settings

All 16 Mozambican midwives working at Beira Central Hospital (Mozambique) and all 18 Italian midwives working at Azienda Ospedaliera-Università di Padova (Italy) participated in the study. Beira Central Hospital is the referral hospital of the province of Sofala, Mozambique. In this hospital, about 5,000 deliveries occur every year and midwives are responsible for immediate postnatal care of all neonates, including resuscitation. Azienda Ospedaliera-Università di Padova is a referral hospital in North-Eastern Italy. In this hospital, about 3,000 deliveries occur every year and midwives are responsible for immediate postnatal care in low-risk deliveries. Both hospitals have the same neonatal resuscitation algorithm (NRP) apart from intubation and medications (7).

### Assessment of Neonatal Resuscitation

Participants were asked to evaluate the performance of a neonatal resuscitation by reviewing the video recording.

The video showed an African full-term newborn with severe asphyxia requiring full resuscitation (initial steps, bag-mask ventilation and chest compressions), which was performed by a local midwife at Beira Central Hospital. This video was chosen from a video collection used in a previous study (4) because it displayed all phases of neonatal resuscitation. The identity of the midwife performing the resuscitation was protected, as the video displayed only the baby and the hands of the resuscitating provider.

Participants were instructed to evaluate the performance using a pre-defined composite score, which was described elsewhere (4). Briefly, two points were awarded for every correct decision and for every procedure that was performed properly. One point was awarded if the intervention was delayed or the technique for a given procedure was inadequate. No points were awarded for indicated procedures that were omitted or for performed procedures that were not indicated. When a step was anticipated due to skipping of the previous step, the first step was scored 0 and the following one was scored 2. When a step was delayed despite skipping the previous step, the first step was scored 0 and the following one was scored 1 (Supplementary Material). This approach allowed discriminating these different scenarios, thus leading to a more detailed score of the entire performance. Points were summed to obtain a total score for each level of resuscitation (initial steps, bag-mask ventilation and chest compressions). Scores by participants were compared with referral scores given by two neonatologists expert in neonatal resuscitation (DT, SC).

### Opinions on Importance of Resuscitation Interventions

Participants were asked to fill out a written form about the importance of each aspect of neonatal resuscitation. The form included 13 items on both technical and non-technical skills, each of them was scored as: 0) not important, 1) fairly important, 2) important, and 3) very important. Scores by participants were compared with referral scores given by two neonatologists expert in neonatal resuscitation (DT, SC).

### Statistical Analysis

Continuous data were expressed as median and interquartile range (IQR), and categorical data as number and percentage. Participant characteristics were compared between Mozambican and Italian midwives using Mann-Whitney-test (continuous data) or Fisher's exact-test (categorical data). Scores by participants were reported as score difference by subtracting the referral score for each item. Score differences were evaluated using Wilcoxon signed rank-test within Mozambican and Italian midwives, and compared between Mozambican and Italian midwives using Mann-Whitney-test. All tests were 2-sided and a *p*-value lower than 0.05 was considered statistically significant. Statistical analysis was performed using R 3.5 (R Foundation for Statistical Computing, Vienna, Austria) (11).

### Ethics Committee Approval

The study was approved by the National Committee of Bioethics (Ref. 315/CNBS/13; November 1, 2013) and by the Minister of Health of the Republic of Mozambique (Ref. 08/GMS/002/2014; January 7, 2014) and by the Hospital Management of the Beira Central Hospital (August 18, 2020), and by the Ethics Committee of University of Padua (Prot. n. 0021324 del 1/4/2020). Before delivery, parents gave their consent to obtain video recordings of neonatal delivery room management and to use the data for scientific purpose (4). Written consent was obtained by participants.

## Results

### Participants

The study included 34 midwives (18 Italians and 16 Mozambican). Participant characteristics are shown in Table 1. Italian midwives were older and more experienced than Mozambican midwives (Table 1). All 18 Italian midwives (100%) and 11 Mozambican midwives (69%) participated in a previous course on neonatal resuscitation (Table 1).

**Table 1:** Participant characteristics.

	**Mozambican midwives**	**Italian midwives**	***p*-value**
	**Median (IQR) or *n* (%)**	**Median (IQR) or *n* (%)**	**-**
*N* participants	16	18	-
Age, years	27 (24; 35)	36 (32; 45)	0.007
Experience in delivery room, months	12 (6; 19)	84 (48; 216)	0.0003
Participation in a previous course on neonatal resuscitation	11 (69%)	18 (100%)	0.02
Time elapsed from the last course, months	8 (6; 12)	4 (3; 12)	0.28
Self-estimation of competence in neonatal resuscitation:			0.45
Poor	1 (6%)	2 (11%)	
Moderate	10 (63%)	7 (39%)	
Good	5 (31%)	9 (50%)	

### Evaluation of Real-Life Neonatal Resuscitation Using Video Review

Table 2 shows the evaluation by Mozambican and Italian midwives (as compared to the evaluation by two experts in neonatal resuscitation) of the performance of a neonatal resuscitation. Both groups overestimated the performance regarding initial steps (*p* = 0.0007 and *p* = 0.004, respectively) and chest compressions (*p* = 0.009 and *p* = 0.009, respectively), with larger overestimation in Mozambican vs. Italian midwives (initial steps *p* = 0.0009 and chest compressions *p* = 0.03). Moreover, Mozambican midwives overestimated the performance regarding bag-mask ventilation (*p* = 0.009).

**Table 2:** Evaluation of the performance of a neonatal resuscitation by reviewing a video showing an African full-term newborn with severe asphyxia requiring full resuscitation (initial steps, bag-mask ventilation and chest compressions), which was performed by a local midwife at Beira Central Hospital.

	**Mozambican midwives vs. reference: score difference**		**Italian midwives vs. reference: score difference**		**Mozambican midwives vs. Italian midwives**
**Aspects of neonatal resuscitation**	**Media*n* (IQR)**	***p*-value**	**Media*n* (IQR)**	***p*-value**	***p*-value**
Initial steps	6 (4; 6)	0.0007	2 (0; 4)	0.004	0.0009
Bag-mask ventilation	1 (0; 3)	0.009	0 (−1; 1)	0.84	0.03
Chest compressions	3 (2; 4)	0.009	1 (0; 2)	0.009	0.03

*The table shows the evaluation by Mozambican and Italian midwives (as compared to the evaluation by two experts in neonatal resuscitation)*.

### Importance of Resuscitation Interventions

**Figure 1:**
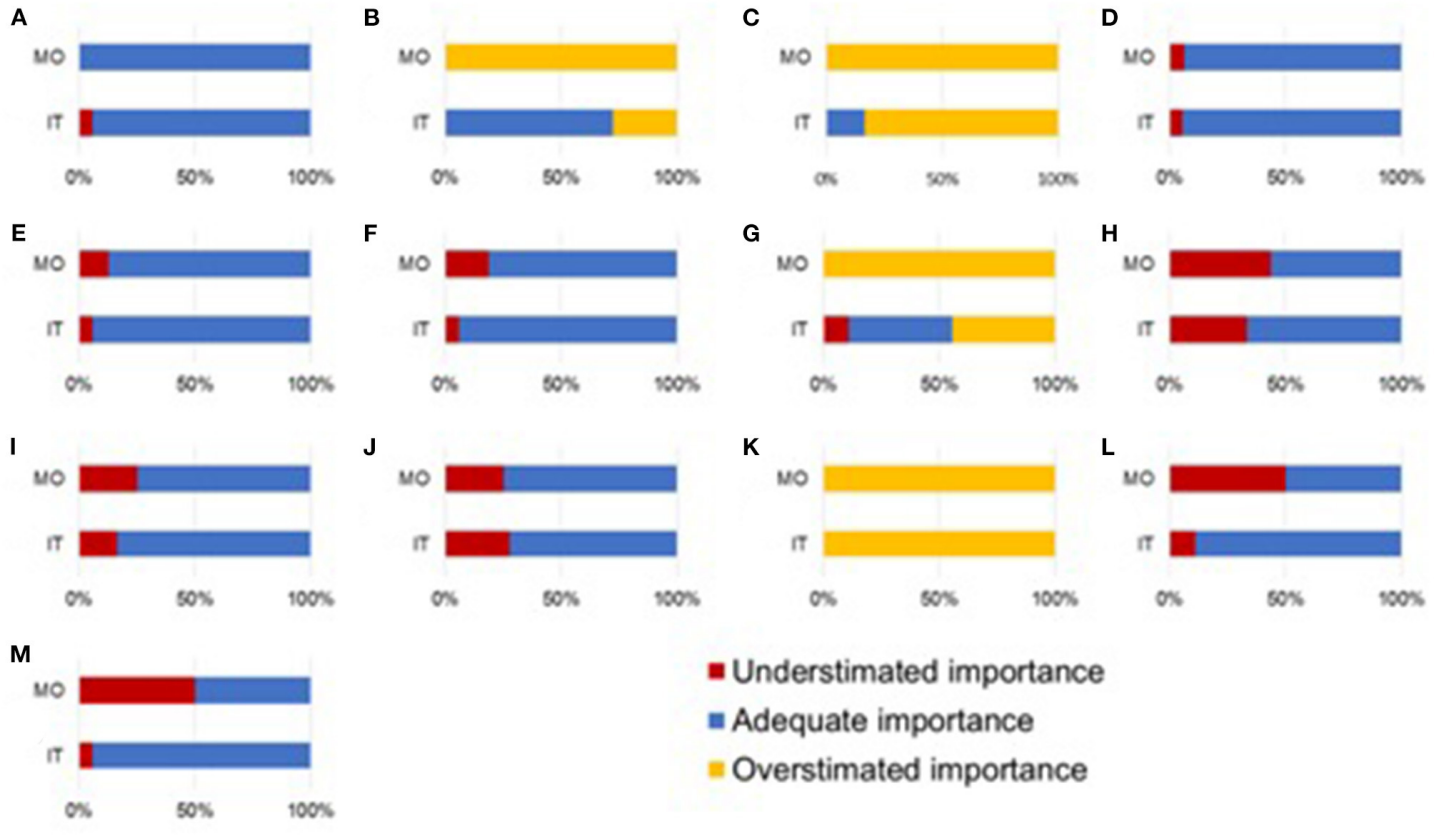
Overlook of importance of resuscitation interventions according to Mozambican (MO) midwives and Italian (IT) midwives (as compared to importance according to two experts in neonatal resuscitation). Resuscitation interventions: **(A)** preparation of equipment, **(B)** weighing the newborn, **(C)** giving high-oxygen concentrations, **(D)** putting the newborn under infant warmer, **(E)** drying the newborn, **(F)** removing wet linen, **(G)** suctioning, **(H)** stimulation, **(I)** heart rate assessment, **(J)** bag-mask ventilation, **(K)** chest compressions, **(L)** interventions based on priorities, and **(M)** adherence to steps of neonatal resuscitations.

Table 3 and Figure 1 report the importance of resuscitation interventions according to Mozambican and Italian midwives (as compared to importance according to two experts in neonatal resuscitation).

**Table 3:** Importance of resuscitation interventions according to Mozambican and Italian midwives (as compared to importance according to two experts in neonatal resuscitation).

	**Mozambican midwives vs. reference:v** ** score differences**		**Italian midwives vs. reference:** **score differences**		**Mozambican midwives vs. Italian midwives**
**Aspects of neonatal resuscitation**	**Median (IQR)**	***p*-value**	**Median (IQR)**	***p*-value**	***p*-value**
Preparation of equipment	0 (0; 0)	NA	0 (0; 0)	0.99	0.38
Weighing the newborn	2 (1; 2)	0.0004	0 (0; 1)	0.04	<0.0001
Giving high-oxygen concentrations	3 (2; 3)	0.0003	2 (1; 2)	0.0005	0.004
Putting the newborn under infant warmer	0 (0; 0)	0.34	0 (0; 0)	0.99	0.46
Drying the newborn	0 (0; 0)	0.99	0 (0; 0)	0.99	0.97
Removing wet linen	0 (0; 0)	0.15	0 (0; 0)	0.99	0.25
Suctioning	2 (1; 2)	0.0003	0 (0; 1)	0.06	<0.0001
Stimulation	0 (-1; 0)	0.01	0 (−1; 0)	0.03	0.65
Heart rate assessment	0 (0; 0)	0.07	0 (0; 0)	0.17	0.64
Bag-mask ventilation	0 (0; 0)	0.07	0 (−1; 0)	0.04	0.87
Chest compressions	2 (1; 2)	0.0003	2 (1; 2)	0.0003	0.52
Interventions based on priorities	−1 (−1; 0)	0.01	0 (0; 0)	0.35	0.01
Adherence to steps of neonatal resuscitations	−1 (−1; 0)	0.006	0 (0; 0)	0.99	0.004

*In the original forms, aspects were scored as 0) not important, 1) fairly important, 2) important, and 3) very important*.

*NA, not available*.

Both groups overestimated the importance of weighing the newborn (*p* = 0.0004 and *p* = 0.04, respectively) and giving high-oxygen concentrations (*p* = 0.0003 and *p* = 0.005, respectively), with larger overestimation in Mozambican vs. Italian midwives (weighing the newborn *p* < 0.0001 and giving high-oxygen concentrations *p* = 0.004).

Both groups overestimated the importance of chest compressions (*p* = 0.0003 and *p* = 0.0003, respectively), with similar magnitude (*p* = 0.52).

Mozambican midwives overestimated the importance of suctioning (*p* = 0.0003).

Both groups underestimated the importance of stimulation (*p* = 0.01 and *p* = 0.03, respectively), with similar magnitude (*p* = 0.65).

Italian midwives underestimated the importance of bag-mask ventilation (*p* = 0.04), while Mozambican midwives underestimated the importance of interventions based on priorities (*p* = 0.01) and adherence to steps of neonatal resuscitations (*p* = 0.006).

Both groups gave adequate importance to preparation of equipment, putting the newborn under infant warmer, drying the newborn, removing wet linen and heart rate assessment (Table 3).

## Discussion

In our study, midwives were prone to overestimate resuscitation performance of their peer regarding initial steps and chest compressions, with more generous evaluation by Mozambican than Italian midwives. Mozambican midwives also overestimated the performance regarding bag-mask ventilation. Furthermore, the importance of some aspects of neonatal resuscitation were overestimated (weighing the newborn, giving high-oxygen concentrations, suctioning, and chest compressions), while others were underestimated (stimulation, interventions based on priorities and adherence to steps of neonatal resuscitations), with some differences between Mozambican and Italian midwives.

The strengths of this study include the use of video recording displaying extensive neonatal resuscitation performed by a local midwife, the participation of all midwives in the two hospitals, and the use of a pre-defined score (4, 12). This study has also some limitations that should be consider when reading the results. First, the number of participants was limited, despite the inclusion of the entire staff. Second, the assessment was limited to only one video, which was chosen to show an extensive neonatal resuscitation. Third, adjustment for multiple testing was not performed due to the exploratory—rather than confirmatory—nature of the study, thus increasing the likelihood of false positive results. Fourth, some aspects of neonatal resuscitation (such as intubation and teamwork) were not investigated because they were not performed at Beira Central Hospital.

Worldwide, neonatal care at birth is entrusted to millions of healthcare providers who must acquire—but also maintain over time—a complex set of technical and non-technical skills (13). Since the most effective educational strategy to ensure and maintain adequate cognitive, technical, and behavioral skills is still unclear (9, 13), identifying weaknesses and misbelieves of healthcare providers can help trainers in selecting educational goals. When reviewing a videorecording of real-life neonatal resuscitation performed by a peer, midwives tended to overestimate the quality of resuscitation procedures, thus revealing a limited perception of the quality of such procedures. Of note, Mozambican midwives were more generous than Italian midwives, which might mirror the difference in experience and education on neonatal resuscitation in the two settings. Despite the implementation of training programs on neonatal resuscitation, our results revealed limited knowledge and awareness of the correct application of the procedures. These findings were consistent with recognized knowledge gaps about the most effective training programs in high-, middle- and low-resource settings (9, 13–15).

To provide a deeper understanding of their evaluation, midwives were also asked to rate the importance of each aspect of neonatal resuscitation. Some aspects (giving high-oxygen concentrations, suctioning, and chest compressions) were considered more relevant than their actual importance, as they have recently received less consideration in international guidelines (8, 13). In addition, weighing the newborn has the potential risk of delaying life-saving interventions (such as initiation of positive pressure ventilation). Adherence to resuscitation guidelines and interventions based on priorities are considered key elements in emergency situations (13, 16) but were considered less important by Mozambican midwives. Giving adequate importance to each aspect of neonatal resuscitation is part of a set of abilities (so-called “behavioral skills”) that are not strictly included in medical knowledge and manual skills, but are required to effectively manage emergency situations (17). Although neonatal resuscitation programs (i.e., NRP and HBB) bestow no considerations about behavioral skills (7, 8), there is a growing interest in developing such skills as vector for enhancing the transfer of theoretical knowledge and skills into clinical practice (9, 17–19). Overall, our findings offer interesting information that could be helpful in targeting goals for training programs on neonatal resuscitation in both high- and low-resource settings. This study also offers a novel idea of testing the knowledge of midwives in order to improve their training. This approach can be implemented in future studies including more videos from different centers and more independent reviewers.

In conclusion, Mozambican and Italian midwives overestimated the performance of a real-life neonatal resuscitation using video recording. When asked to score the importance of some aspects of neonatal resuscitation, they deviated from the scores given by experts in neonatal resuscitation in several important aspects. These findings highlighted the opportunity of focus in on the so-called “behavioral skills” during training programs in order to transfer theoretical knowledge and skills into clinical practice.

## Data Availability Statement

The raw data supporting the conclusions of this article will be made available by the authors, without undue reservation.

## Ethics Statement

The study was approved by the National Committee of Bioethics (Ref. 315/CNBS/13; November 1, 2013) and by the Minister of Health of the Republic of Mozambique (Ref. 08/GMS/002/2014; January 7, 2014) and by the Hospital Management of the Beira Central Hospital (August 18, 2020), and by the Ethics Committee of University of Padua (Prot. n. 0021324 del 1/4/2020). Written informed consent to participate in this study was provided by the participants' legal guardian/next of kin.

## Author Contributions

FC performed the statistical analysis, contributed to data interpretation, writing of the manuscript, and critically reviewed the manuscript. SC, MB, and MM performed the literature review, collected the data in Mozambique, contributed to data interpretation, drafted the initial manuscript, and critically reviewed the manuscript. GB performed the literature review, collected the data in Italy, contributed to data interpretation, and critically reviewed the manuscript. AS, AT, and BC contributed to the collection of data in Mozambique, contributed to data interpretation and critically reviewed the manuscript. GP and DT conceptualized the study, contributed to data interpretation, and writing of the manuscript and critically reviewed the manuscript. All authors approved the final manuscript as submitted and agree to be accountable for all aspects of the work.

## Conflict of Interest

The authors declare that the research was conducted in the absence of any commercial or financial relationships that could be construed as a potential conflict of interest.
