# Supplementary material for: Midwives' Evaluation of a Neonatal Resuscitation in High- and Low-Resource Settings
Source: Front Pediatr. 2021 Mar 9;9:644308. doi: 10.3389/fped.2021.644308 (PMC7985172; doi:10.3389/fped.2021.644308)
Supplement: Supplementary file 1 [file Table_1.DOCX]

**Supplementary material. Scoring system**

Instructions:

Two points are awarded for every correct decision and for every procedure that is performed properly.

One point is awarded if the intervention is delayed or the technique for a given procedure is inadequate.

No points are awarded for indicated procedures that are omitted or for performed procedures that are not indicated.

When a step is anticipated due to skipping of the previous step, the first step is scored no points and the following one is scored 2 points. When a step is delayed despite skipping the previous step, the first step is scored no points and the following one is scored 1 point.

|  |  | Points (0-2) |
| --- | --- | --- |
| **Initial steps** | Check material (suction system, bag, mask, oxygen supply) |  |
|  | Body positioned correctly |  |
|  | Head positioned correctly |  |
|  | Suctioning (if indicated) |  |
|  | Dry the baby |  |
|  | Remove wet linen |  |
|  | Neonate uncovered under infant warmer |  |
|  | Stimulation |  |
|  | HR assessment (stethoscope or or umbilical pulse) |  |
|  | **Total** |  |
| **Ventilation** | Start PPV in room air |  |
|  | Correct face mask positioning |  |
|  | Ventilatory rate at 40-60 breaths/min |  |
|  | Adequate chest rise |  |
|  | HR assessment after 30 sec (stethoscope or umbilical pulse) |  |
|  | **Total** |  |
| **Chest compressions** | Correct method (2 fingers/2 thumbs) |  |
|  | Correct frequency and depth |  |
|  | Correct ventilatory frequency |  |
|  | Correct CC/ventilation ratio (3/1) |  |
|  | HR assessment after 60-90 sec (stethoscope or umbilical pulse) |  |
|  | **Total** |  |
